# Antimicrobial Activity of Protamine-Loaded Calcium Phosphates against Oral Bacteria

**DOI:** 10.3390/ma12172816

**Published:** 2019-09-02

**Authors:** Masashi Fujiki, Kodai Abe, Tohru Hayakawa, Takatsugu Yamamoto, Mana Torii, Keishi Iohara, Daisuke Koizumi, Rie Togawa, Mamoru Aizawa, Michiyo Honda

**Affiliations:** 1 Department of Applied Chemistry, School of Science and Technology, Meiji University, 1-1-1, Higashimita, Tama-ku, Kawasaki 214-8571, Kanagawa, Japan; 2 Department of Dental Engineering, Tsurumi University School of Dental Medicine, 2-1-3, Tsurumi, Tsurumi-ku, Yokohama 230-8501, Kanagawa, Japan; 3 Department of Operative Dentistry, Tsurumi University School of Dental Medicine, 2-1-3, Tsurumi, Tsurumi-ku, Yokohama 230-8501, Kanagawa, Japan; 4 Department of Removable Prosthodontics, Tsurumi University School of Dental Medicine, 2-1-3, Tsurumi, Tsurumi-ku, Yokohama 230-8501, Kanagawa, Japan; 5 Central Research Institute, Maruha Nichiro Corporation, 16-2, Wadai, Tsukuba 300-4295, Ibaraki, Japan

**Keywords:** protamine, antimicrobial peptide, dicalcium phosphate anhydride, dental material, *Streptococcus mutans*, biofilm formation

## Abstract

Protamine is an antimicrobial peptide extracted from fish. In this study, we loaded protamine onto dicalcium phosphate anhydride (DCPA), a dental material. Protamine was loaded by stirring DCPA into a protamine solution. To explore the antimicrobial activity of the materials, we cultivated *Streptococcus mutans* on fabricated discs for 24 h. When *S. mutans* was cultivated on the discs under no sucrose conditions, the loaded protamine was not released, and the ratio of dead bacteria increased on the surface of P (125) DCPA (half of the saturated level of protamine (125 ppm protamine) was loaded). Aside from P (500) DCPA (saturated level of protamine was loaded), some protamine was released, and the number of planktonic bacteria in the supernatant decreased. Using medium containing 1% sucrose, the release of protamine was promoted from P (125) DCPA due to lowered pH. However, lowering of the pH decreased the antimicrobial activity of protamine. On the other hand, P (500) DCPA released protamine before the pH was lowered, and biofilm formation was inhibited. The loaded protamine expressed antimicrobial activity, both on the surface of the materials and in the surrounding environment. The interaction of loaded protamine with calcium phosphates could promote the application of protamine in the dental field.

## 1. Introduction

Protamine is an arginine-rich polycationic protein extracted from the sperm cells of vertebrates, including fish such as salmon [1]. It has been reported that protamine has antimicrobial effects against a wide range of bacteria. In particular, its antimicrobial properties are more effective against gram-positive bacteria than they are against gram-negative bacteria [2]. Electrostatic interaction with the cell envelope is considered to be the initial antibacterial mechanism of most cationic peptides, such as magainin [3]. Their hydrophobic portions then interact with the hydrophobic components of the membrane and cause membrane destruction. This characteristic mechanism of action enables antimicrobial agents to avoid the common resistance mechanisms observed for antibiotics [4]. In addition to its antimicrobial effects, protamine also has the advantage of having low cytotoxicity. Biocompatibility testing using mice and rats showed that protamine is highly safe for use in live organisms. Protamine has therefore already been utilized in the food industry, such as in preservatives.

Due to the antimicrobial activity and low toxicity of protamine, we applied protamine to dental materials. Among these materials, we focused on dicalcium phosphate anhydride (DCPA; CaHPO_4_), which is generally mixed with toothpaste for cleaning teeth. DCPA is used, not only as a cleaning agent, but also as a tubular occluding material to suppress dentin hypersensitivity (DH). In fact, DCPA has been used in some desensitizers, showing great potential for reducing dentin permeability [5]. DH caused by exposure of the peripheral termination of the dentinal tubules is a fairly common and persistent problem among the general population [6,7]. 

The opened dentinal tubules are directly immersed in oral fluid, e.g., saliva, and gingival crevicular fluid, which contains a large number of bacteria [6]. Owing to a lower degree of mineralization than the enamel, exposed dentin tends to be more susceptible to dentin caries caused by oral bacteria. Contemporary desensitizing therapy via tubular occlusion can cut off the pathway of bacteria invading the dentin tubules [8]. However, these therapies do not aim to inhibit bacterial growth. The local acidic microenvironment induced by bacterial metabolism may hamper the efficacy and longevity of desensitizing. Therefore, desensitizing therapy that includes an inhibition of oral bacterial growth may be a more promising therapeutic avenue. 

The aim of this study is to evaluate the efficacy of protamine-loaded DCPA (P-DCPA) on the growth and biofilm formation of *Streptococcus mutans* (*S. mutans*) in vitro. *S. mutans* is one of the many etiological factors of dental caries and can rapidly form cariogenic biofilms via dietary sucrose [9]. Studies on the antimicrobial behavior of protamine loaded on calcium phosphates, such as DCPA, is promising for promoting the application of protamine to the dental field.

## 2. Materials and Methods

### 2.1. Preparation of DCPA Powders

Ten grams of commercially-available DCPA powders (Taihei Chemical Industrial Co., Ltd., Osaka, Japan) was ground using a planetary mill (Pulverisette 6, Fritch, Germany) for 180 min at a rotation rate of 300 rpm in a ZrO_2_ pot using ZrO_2_ beads with 10 mm diameter in 40 cm^3^ of ultrapure water. After ball-milling, the slurry was filtered and freeze-dried for 24 h. 

### 2.2. Adsorption of Protamine to DCPA Powders and Fabrication of Discs

Protamine hydrochloride was kindly supplied by Maruha Nichiro Corporation. Protamine solutions (0, 125, 500 μg·cm^−3^) were diluted by ultrapure water. Freeze-dried DCPA powders (1.5 g) was added into a protamine solution (45 cm^3^) and rotated with a constant speed of 60 rpm for 48 h at room temperature. The samples were centrifuged for 15 min at 8000 rpm, and supernatants were removed. Powders were washed with ultrapure water 3 times and freeze-dried. The resulting powders were uniaxially compressed at 20 MPa to form compacts (ϕ = 15 mm, thickness = 2 mm) for antimicrobial evaluation. Hereafter, powders loaded with protamine are denoted by P (x) DCPA; x represents the concentration of protamine. 

### 2.3. Characterization of Protamine-Loaded DCPA

The amounts of loaded-protamine were measured by HPLC analysis (Prominence, SHIMADZU, Kyoto, Japan). In brief, 100 mg of powder was added to 0.45 cm^3^ of 1N-HCl solution and stirred for 1 h. The samples were centrifuged for 2 min at 13000 rpm and supernatant was diluted twice by ultrapure water. Diluted supernatants were filtered with a membrane filter (<0.45 μm), and the filtrates were assayed for HPLC analysis. 

The particle size distribution was measured using a laser diffraction particle size analyzer (SALD-7100, SHIMADZU, Kyoto, Japan) in distilled water at a range of 0.01–300 μm. Before measurement, samples were homogenized for 15 min.

The zeta potential of powders was measured using a laser-doppler velocimeter (ELS-6000, Otsuka Electronics, Osaka, Japan) in 10 mM NaCl solution at 25 °C. The zeta potential for each sample was calculated from the measured value of the electrophoretic mobility. 

### 2.4. Microorganisms

*Streptococcus mutans* (*S. mutans*) was used for the evaluation of antimicrobial testing. *S. mutans* is one of the main etiological agents of dental caries and is a well known biofilm-forming oral bacterium. Strain of *S. mutans*, purchased from NBRC (NBRC 13955, Chiba, Japan), was used in the study. The strains were grown in a Trypticase Soy Yeast Extract Medium (TSYE media) in an anaerobic jar (Mitsubishi gas chemical, Tokyo, Japan) at 37 °C. The cultures were stored at −80 °C in medium containing 25% glycerol.

In this study, we used TSYE media prepared to 1/10 concentration to cultivate *S. mutans*. In the liquid culture media, protamine reacts with the components [2,10]. 

### 2.5. Antibacterial Activity of Protamine-Loaded DCPA Discs against S. mutans 

*S. mutans* were grown on each disc (P (0) DCPA, P (125) DCPA, P (500) DCPA) set on a 24 well plate. Each well in the plate was inoculated with 1.5 cm^3^ of bacterial suspension (4 × 10^6^ CFU·cm^−3^) in 1/10 concentration TSYE media. After incubation anaerobically at 37 °C for 24 h, supernatants were removed and the discs were washed twice with Phosphate Buffered Saline (PBS). 

The pieces of each disc were stained using the LIVE/DEAD^®^ BacLight^TM^ Bacterial Viability Kit (Thermo, Waltham, MA, USA) for 15 min in the dark. Propidium iodide (PI) specifically labels the dead cells with damaged cytoplasmic membranes in red, whereas live cells are labeled in green by SYTO^®^9. The stained bacteria were observed by fluorescence microscope (BZ-X710, KEYENCE, Osaka, Japan).

To observe the morphology of bacteria attached to the discs, other pieces of each disc were fixed with 2.5% glutaraldehyde overnight at 4 °C. Fixed discs were freeze-dried overnight and coated with gold. Each sample was observed with SEM (VE9800, KEYENCE, Osaka, Japan) at 10 kV. 

As for the removed supernatants, planktonic bacteria were quantified by colony count, and free protamine concentration was determined by Bradford assay kit (Fujifilmwako). pH value was measured by pH meter (COMPACT pH METER, HORIBA, Kyoto, Japan). Further calcium concentration was measured by Inductively Coupled Plasma-Atomic Emission Spectrometry (ICP-AES; SPS7800, Seiko Instruments Inc., Chiba, Japan). 

### 2.6. Biofilm Formation on the Protamine-Loaded DCPA Disc

Biofilms were formed on each disc (P (0) DCPA, P (125) DCPA, P (500) DCPA) set on a 24 well plate. Each well in the plate was inoculated with 1.5 cm^3^ of bacterial suspension (4 × 10^6^ CFU·cm^−3^) in 1/10 concentration TSYE media including 1% sucrose. After incubation anaerobically at 37 °C for 24 h, supernatants were removed, and the discs were washed twice with PBS. 

The bacterial cells were labeled with 5.0 μM SYTO^®^9 and exopolysaccharides (EPS) were labeled with 1.0 μM Alexa Fluor 594 dextran conjugate (Thermo, Waltham, MA, USA). Fluorescently labeled dextran serves as a primer for *Gtfs* and can be incorporated during EPS matrix synthesis [11]. Planktonic bacteria, protamine concentration, pH value, and calcium concentration in the supernatants were also measured. 

### 2.7. Effect of pH against Antimicrobial Activity of Protamine

The antimicrobial effects of protamine in different pH conditions was tested by colony count. The pH of each media was adjusted (pH 4, 5, 6, and 7) using 1M HCl. An overnight culture of *S. mutans* was diluted to 4 × 10^6^ CFU·cm^−3^ using TSYE media (pH 4, 5, 6, and 7) prepared to 1/10 concentration. Protamine solution (0.15 cm^3^) (final concentration: 0, 250 μg·cm^−3^), diluted by ultrapure water, and 1.35 cm^3^ of bacterial suspension were added to a 24 well plate. After incubation for 24 h at 37 °C, the number of bacteria in the suspensions was quantified by colony count. 

### 2.8. Statistical Analysis

All the data were statistically analyzed to determine the mean and the standard deviation of the mean. The Student’s *t*-test was performed with a significant level of *p* < 0.05. 

## 3. Results

### 3.1. Characterization of Protamine-Loaded DCPA

To obtain protamine-loaded DCPA powders, DCPA powders were immersed in various concentrations of protamine solution. The adsorption of protamine to the DCPA powders increased with protamine concentration, and reached a plateau at over 500 μg·cm^−3^ (Figure S1). In this study, we used three kinds of protamine-loaded DCPA powders, and their properties are shown in Table 1. The saturated level (500) and half of the saturated level (125) of protamine loaded-DCPA powders are denoted as P (500) DCPA and P (125) DCPA, respectively. The zeta potential of DCPA was negatively charged (−22.34 mV). However, that of protamine-loaded DCPA (P (125) DCPA and P (500) DCPA) was positively charged and increased with protamine concentration. These results suggest that protamine, a cationic peptide, adsorb DCPA powders by electrostatic interaction. As previously reported, the adsorption of basic protein onto a hydroxyapatite disk enhanced or had no effect on bacterial adherence, whereas the adsorption of an acidic protein reduced adherence [12]. Therefore, loading protamine on the DCPA powders did not affect the adherence to the bacterial surface. As for the median size of the powders, there was no significant difference among the three samples. 

### 3.2. Antibacterial Activity of P-DCPA Discs against S. mutans

**Figure 1:**
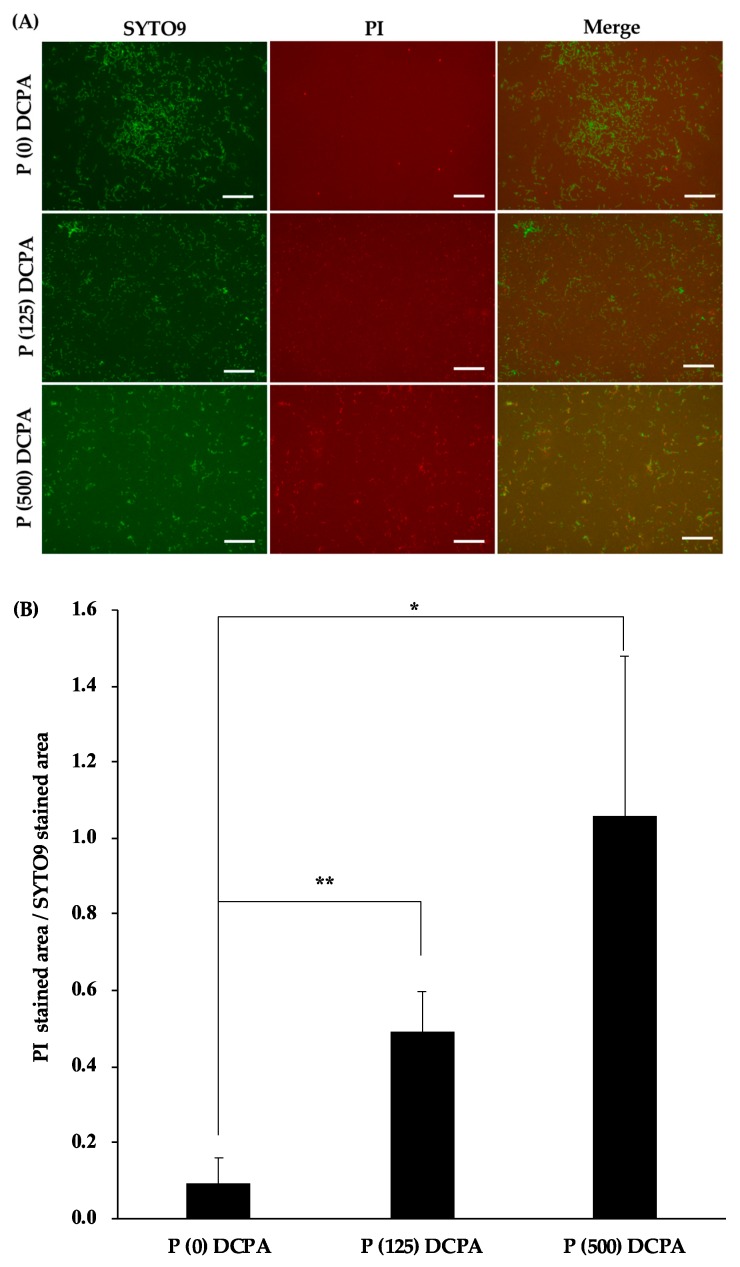

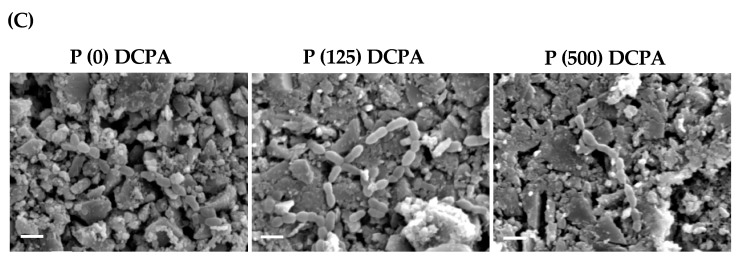
Antimicrobial activity of protamine-loaded DCPA fabricated discs. *S. mutans* (4 × 10^6^ CFU·cm^−3^) were incubated in 1/10 concentration of media with each disc (P (0) DCPA, P (125) DCPA, P (500) DCPA) for 24 h. Error bars indicate standard error of the mean (*n* = 3). The asterisks show * *p* < 0.05, ** *p* < 0.01 by Student’s *t*-test. (**A**) The bacteria attached to the surface of each disc were stained with SYTO^®^9 and PI. Live bacteria appear green and dead bacteria appear red. Bars represent 20 μm. (**B**) The area stained by SYTO^®^9 and PI was analyzed by image analysis. This figure indicates the ratio of dead bacteria to live bacteria. (**C**) SEM images of the bacteria attached to each disc surface. Bars represent 1.0 μm.

To evaluate the antimicrobial activity of protamine-loaded DCPA, *S. mutans* were cultivated on the fabricated discs. The images of the attached bacteria on each disc (P (0) DCPA, P (125) DCPA, and P (500) DCPA) are shown in Figure 1A. Live bacteria stained with SYTO^®^9 showed green fluorescence, whereas dead bacteria stained with propidium iodide (PI) showed red fluorescence. Compared with the control disc (P (0) DCPA), both protamine-loaded DCPA discs affected cell viability. The ratio of dead bacteria/live bacteria increased with an increase in the amount of loaded protamine (Figure 1B). Furthermore, to examine the microstructure of the bacteria on protamine-loaded DCPA discs, the bacterial morphology was observed by SEM (Figure 1C). There were no major abnormalities observed in the bacterial morphology of these samples. 

**Figure 2:**
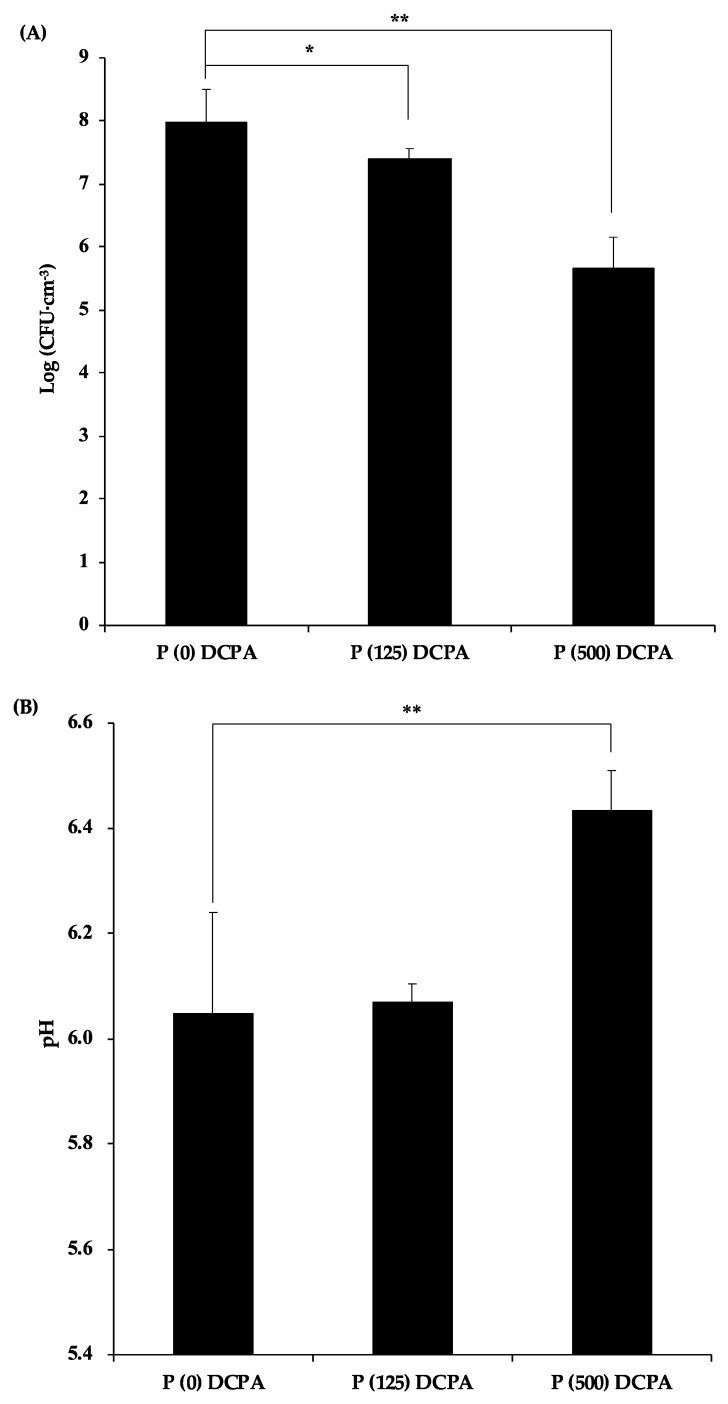

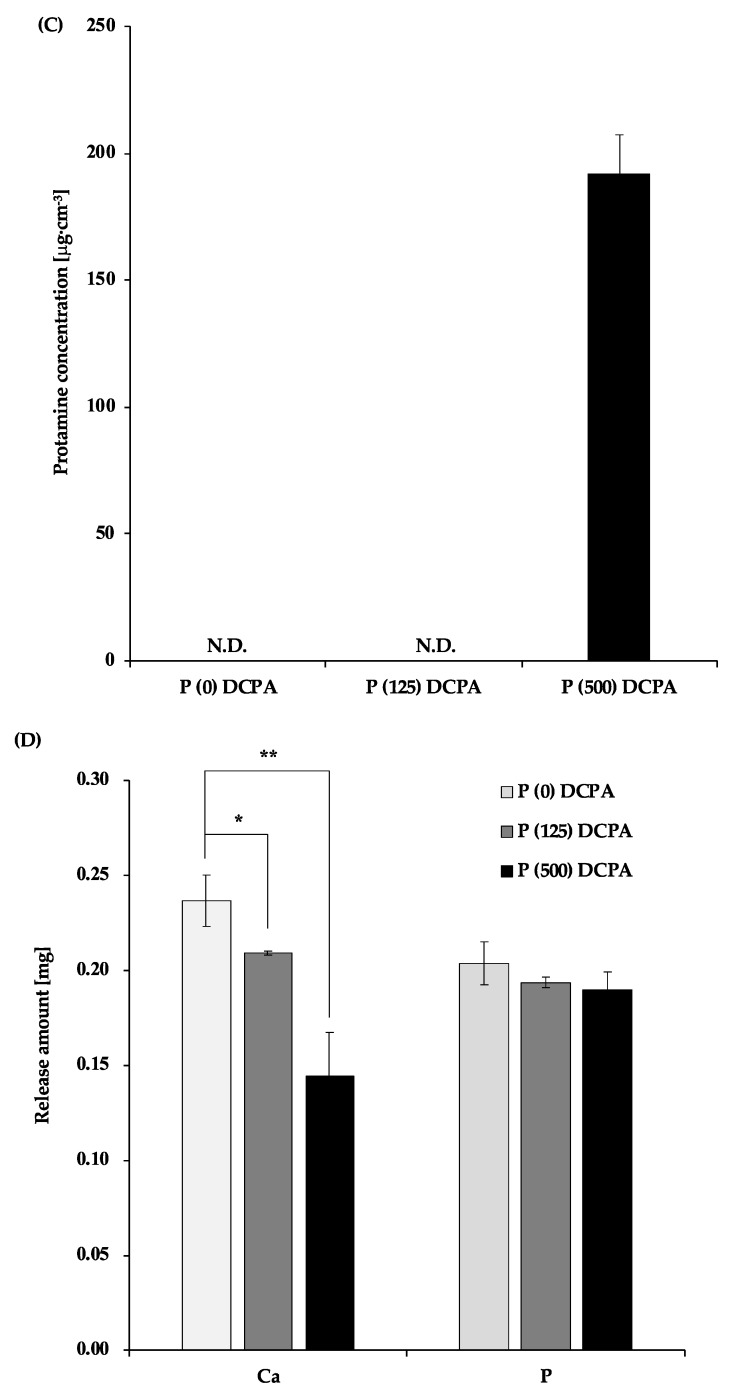
Analysis of the supernatants of fabricated discs. The supernatants of the discs were explored after incubation of *S. mutans* in culture without sucrose for 24 h. Error bars indicate the standard error of the mean (*n* = 3–6). The asterisks show * *p* < 0.05, ** *p* < 0.01 by Student’s *t*-test. (**A**) *S. mutans* cells in supernatant were evaluated by colony counting on an agar plate at 48 h, post-infection. (**B**) The pH value of the supernatant was measured by a pH meter. (**C**) Protamine concentration in supernatant was quantified by Bradford assay. (**D**) Calcium and phosphate release from the discs were quantified by ICP-AES.

Next, to explore the mechanism of antimicrobial activity of protamine-loaded DCPA, the number of planktonic bacteria in the supernatants was measured (Figure 2A). The number of planktonic bacteria in supernatants decreased with an increase in loaded protamine. The pH value of the supernatants was in the range of 6.0–6.5 in these samples (Figure 2B). Approximately 200 μg·cm^−3^ of protamine was detected in the supernatant of the P (500) DCPA disc, whereas we could not detect protamine in the supernatant of the P (125) DCPA disc (Figure 2C). 

We also measured the amount of calcium and phosphorus ions released from the discs (Figure 2D). As a result, the amount of calcium ions released from the discs decreased with increasing loaded protamine. On the other hand, no significant changes in the amount of released phosphorous ions were observed among the three samples. 

These results indicate that bacterial growth was inhibited on the P (125) and P (500) DCPA discs, and that the pH of the supernatants was sustained at around 6 when *S. mutans* were cultivated without sucrose.

We could not observe the release of protamine from P (125) DCPA nor a significant reduction of planktonic bacteria in the supernatants. In contrast with the planktonic bacteria in the supernatants, the surface of the P (125) DCPA disc exhibited antimicrobial activity. As for P (500) DCPA, about 10% of the loaded protamine was released from the disc, and significant antimicrobial effects were observed in the supernatant. Furthermore, antimicrobial activity on the disc surface was confirmed. 

### 3.3. Biofilm Formation on the P-DCPA Discs

**Figure 3:**
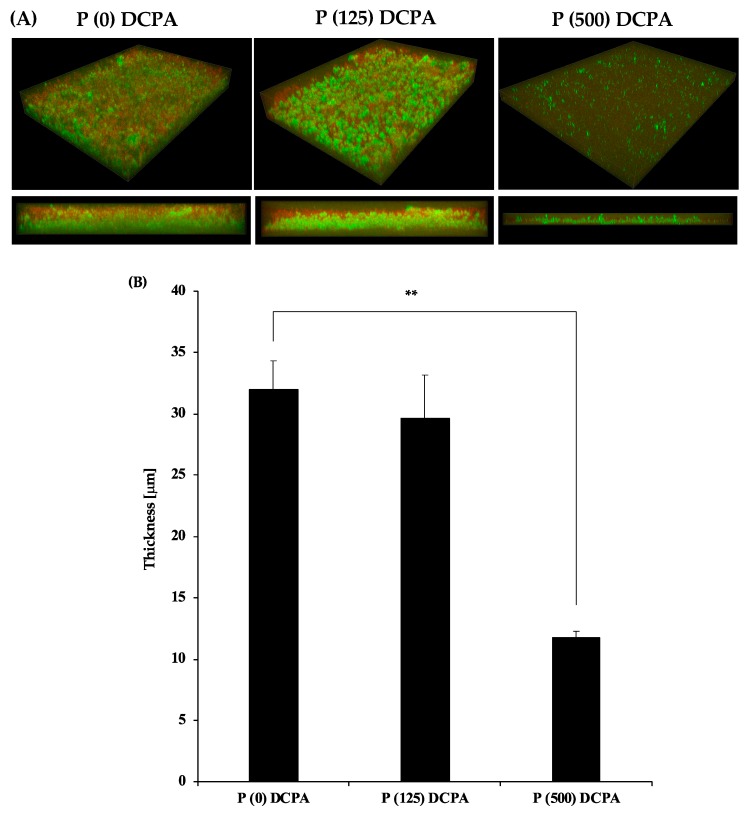

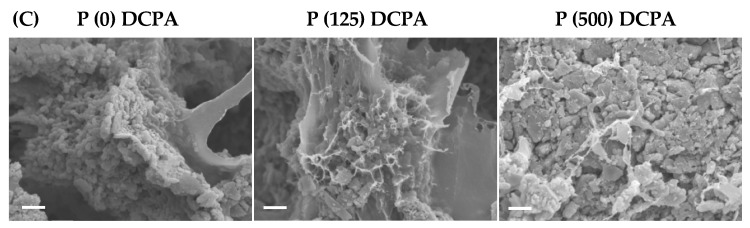
Antimicrobial activity of protamine-loaded DCPA fabricated discs in culture conditions with 1% sucrose. *S. mutans* (4 × 10^6^ CFU·cm^−3^) were incubated in 1/10 concentration of media containing 1% sucrose for each disc (P (0) DCPA, P (125) DCPA, P (500) DCPA) for 24 h. Error bars indicate the standard error of the mean (*n* = 3). The asterisks show ** *p* < 0.01 by Student’s *t*-test. (**A**) Three-dimensional architecture and cross section of the biofilm on the disc stained by SYTO^®^9 and dextran conjugates. Live bacteria appear green and extracellular polysaccharides (EPS) appear red. (**B**) Thickness of the biofilm measured by image analysis. (**C**) SEM image of the biofilm on the disc surface. Bars represent 2.0 μm.

*S. mutans* synthesize adhesive glucans from sucrose, and glucans mediate the firm adherence of its cells to the tooth surface [9,13]. To mimic an in vivo environment, we cultivated *S. mutans* in normal medium with 1% sucrose and examined the antimicrobial activity of protamine-loaded DCPA. The three-dimensional images of the biofilm stained by SYTO^®^9 and dextran conjugates are shown in Figure 3A. Live bacteria stained with SYTO^®^9 showed green fluorescence, whereas extracellular polysaccharides (EPS) stained by dextran conjugates showed red fluorescence. Compared with the P (0) DCPA and P (125) DCPA discs, there was no difference in biofilm thickness and EPS production. On the other hand, P (500) DCPA inhibited biofilm formation, particularly EPS production (Figure 3A,B). Live/dead bacterial staining also showed a significant reduction in live bacteria on the P (500) DCPA disc (Figure S2). To explore its bacterial morphology in detail, *S. mutans* were observed in each disc by SEM. As a result, we identified fibrous morphology concomitant with bacteria on the P (125) DCPA and P (500) DCPA disc surfaces (Figure 3C).

**Figure 4:**
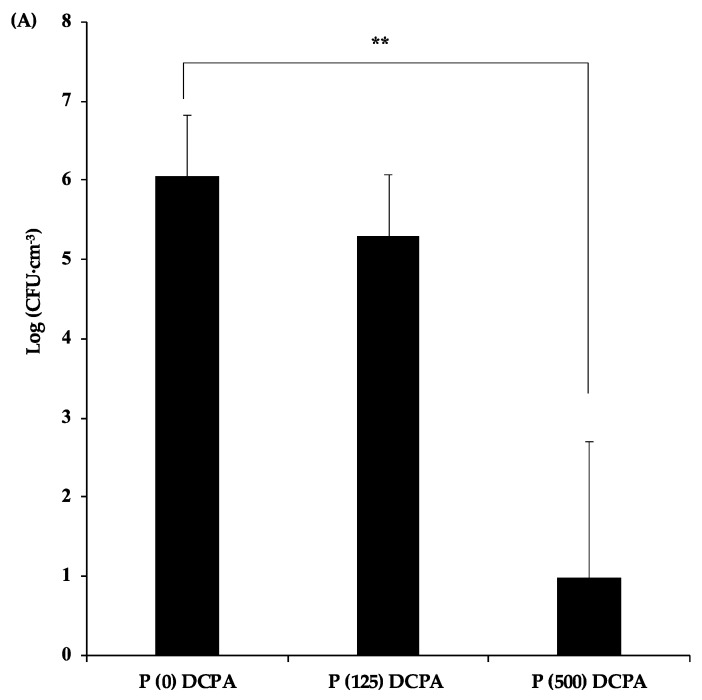

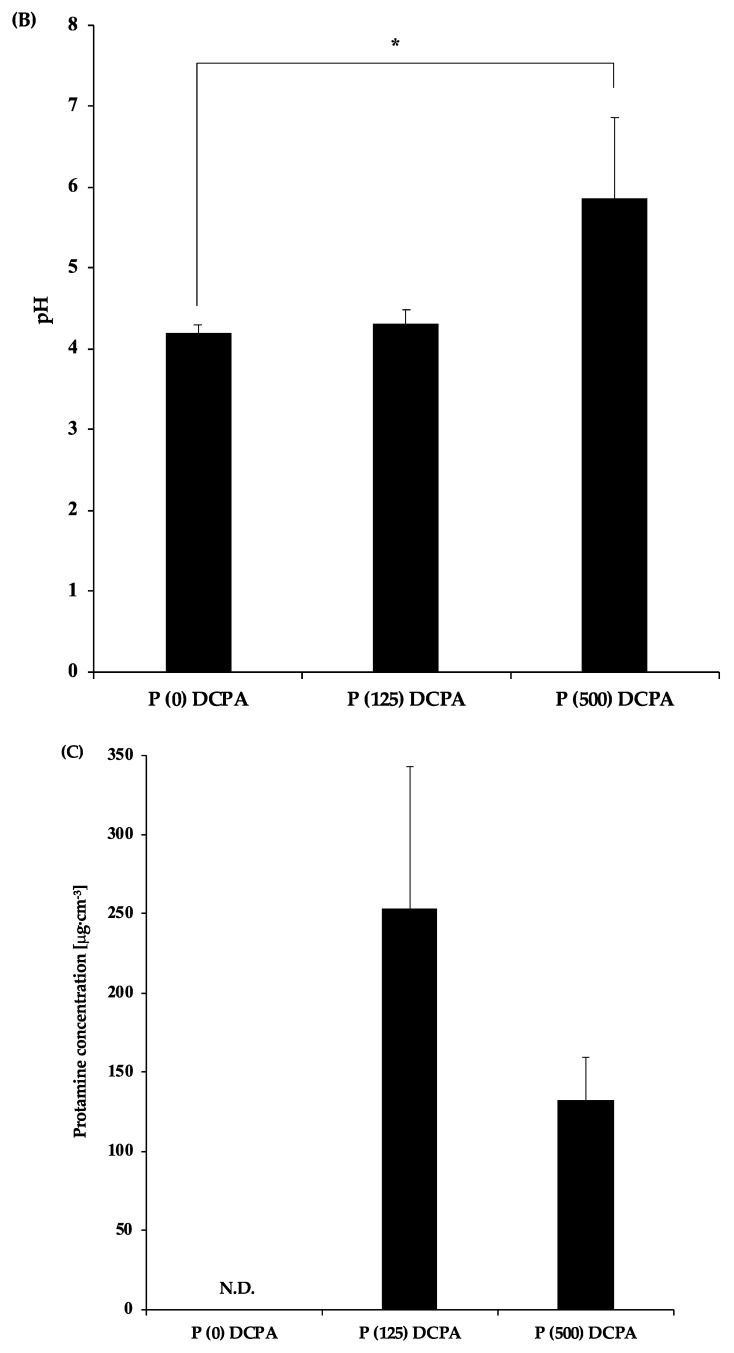

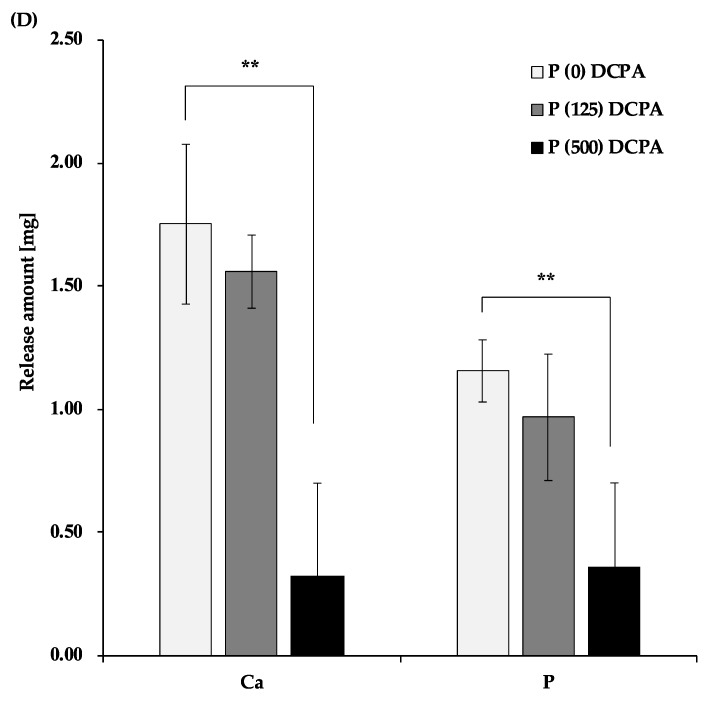
Analysis of the supernatants of the fabricated discs. The supernatants of the discs were explored after incubation of *S. mutans* in culture containing 1% sucrose for 24 h. Error bars indicate the standard error of the mean (*n* = 3). The asterisks show * *p* < 0.05, ** *p* < 0.01 by Student’s *t*-test. (**A**) *S. mutans* cells in supernatant were evaluated by colony counting on an agar plate at 48 h post-infection. (**B**) The pH value of the supernatant was measured by a pH meter. (**C**) Protamine concentration in supernatant was quantified by Bradford assay. (**D**) Calcium and phosphate release from the discs were quantified by ICP-AES.

To clarify the mechanism of antimicrobial activity of protamine-loaded DCPA against biofilm formation, the planktonic bacteria in the supernatants were examined. The number of planktonic bacteria in the supernatant of the P (500) DCPA disc was lower than other samples (Figure 4A). 

In addition, the pH values in the supernatant of the P (500) DCPA disc (pH 5.9) were significantly higher than those of the P (0) DCPA (pH 4.2) and P (125) DCPA (pH 4.3) discs (Figure 4B). Due to higher supernatant pH, the amount of calcium and phosphorus ions released from the P (500) DCPA disc was the lowest among the three samples (Figure 4D). Interestingly, the number of planktonic bacteria in the supernatant of the P (125) DCPA disc did not decrease compared with the control, although there was approximately 250 μg·cm^−3^ protamine in the supernatant (Figure 4C). 

In a cultivation environment containing 1% sucrose, the pH of the supernatants dropped to around 4, with biofilm formation. For the P (500) DCPA sample, the disc released protamine before lowering the pH, and the released protamine expressed antimicrobial activity in the supernatant. In the case of P (125) DCPA, the lowering of the pH with biofilm formation promoted the release of protamine from the disc with dissolution of the DCPA powders. However, there was no significant decrease in the number of planktonic bacteria when protamine was released. This result suggests that the change in pH influenced the antimicrobial activity of protamine in the sample supernatants. 

### 3.4. The Effect of pH against Antimicrobial Activity of Protamine

**Figure 5:**
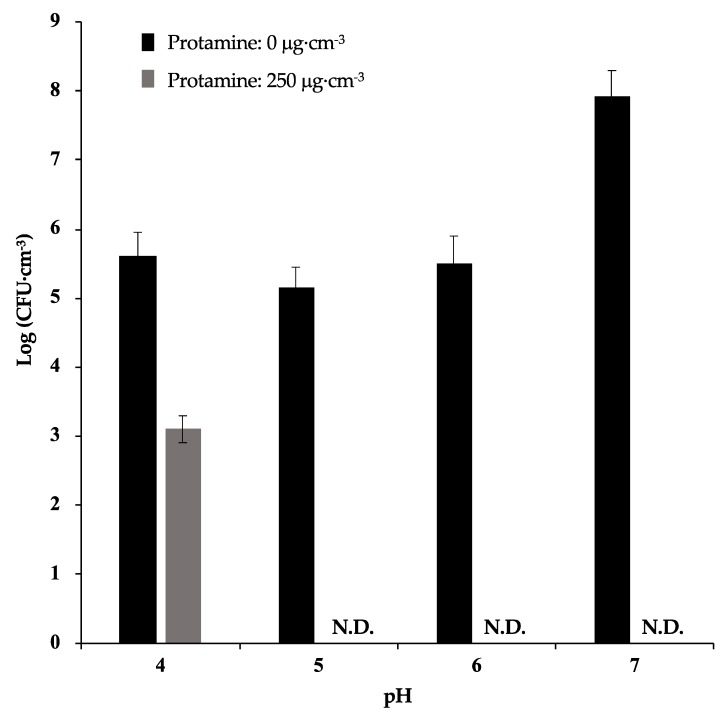
Effect of pH on antimicrobial activity of protamine. *S. mutans* (4 × 10^6^ CFU·cm^−3^) were cultivated under different pH conditions for 24 h. After incubation, the number of bacteria was measured by colony counting. Error bars indicate the standard error of the mean (*n* = 3).

To investigate the effect of pH against the antimicrobial activity of protamine, we incubated *S. mutans* with 250 μg·cm^−3^ protamine under different pH conditions (pH 7, 6, 5, and 4). After incubation for 24 h, no bacteria were detected when treated with protamine in a culture environment of pH 7, 6, and 5 (Figure 5). However, in the culture media with pH 4, there was only an approximate reduction of 10^2^ in protamine compared with the control (no protamine). These results suggest that a decrease in pH reduces the antimicrobial activity of protamine.

## 4. Discussion

Several desensitizers have been introduced for hypersensitivity treatment. These treatments are mainly categorized as tubular occlusion and blockage of nerve activity [14]. Above all, calcium phosphates containing desensitizers have evoked considerable interest due to their biocompatible properties [5,15]. Among the many kinds of calcium phosphate materials, DCPA has been used in some desensitizers, such as Teethmate Desensitizer (Kuraray Noritake Dental Inc., Tokyo, Japan). Desensitizers containing DCPA occlude exposed dental tubules and reduce dentin permeability while transforming to hydroxyapatite. Mixing with aqueous solutions leads to DCPA dissolution, supplying calcium and phosphate ions. The calcium and phosphate ions are precipitated again on the surface of DCPA in the form of hydroxyapatite [16,17]. However, the lactic acid produced by oral bacteria such as *S. mutans* dissolves hydroxyapatite [18]. Bacterial growth around dental materials may therefore reduce the effects of desensitization by dissolution of calcium phosphate components.

In this study, we have prepared DCPA powders by loading protamine as an antimicrobial agent. Protamine generally consists of 20 arginine molecules from a total of 30 amino acids [19]. Due to its high arginine content, protamine has a highly positive charge and can adsorb to negatively charged DCPA. Electrostatic attraction acts as the driving force for protein adsorption on ionic surfaces [20,21]. In the past, applying antimicrobial peptides (AMPs) as a coating on material surfaces has been attempted to provide antimicrobial activity. This has included immobilizing AMPs onto the surface of biomaterials through chemical coupling, such as silanization [22]. However, these coupling procedures are usually sophisticated and difficult to control. On the other hand, in our study, we loaded protamine onto materials by stirring DCPA into a protamine solution. The adsorption efficiency of protamine was also high, providing additional simplicity to the process. Before the adsorption of protamine reached saturation, free protamine in the solution was not detected (Figure S1). 

To evaluate the antimicrobial activity of protamine-loaded DCPA, we cultured *S. mutans* on the fabricated discs. In the case of P (125) DCPA, which loaded half of the saturated level of protamine, the ratio of dead bacteria on the surface of the disc increased, and protamine was not released from the materials (Figure 1B and Figure 2C). These results demonstrated that loaded protamine has desirable antimicrobial activity against *S. mutans* on material surfaces. The antimicrobial activity of the material surface could also lead to the death of planktonic bacteria in the solution [23]. However, when biofilm formation was promoted, the antimicrobial activity of the material surfaces was considered deactivated (Figure 3A,B). During biofilm formation, some killed bacteria may remain on the antimicrobial active surface, and new approaching bacteria can adhere to these killed bacteria [22]. Biofilm formation on the disc surface led to a drop in the pH of the culture environment and resulted in DCPA dissolution. Dissolution of DCPA made it easy to release loaded protamine. However, the antimicrobial activity of protamine reduced upon pH decrease (Figure 5), and the number of bacteria in the supernatants did not significantly decrease compared with the control (Figure 4A). Previous studies have shown that the amount of protamine binding to the surface of cells depends on the pH value [10]. The amount of protamine adsorbed to the cell surface increased with increasing pH [24]. Additionally, an increase in the concentration of calcium ions may reduce the antimicrobial activity of protamine. Divalent cations such as Ca^2+^ may shield negatively charged phospholipids in the cytoplasmic membrane and peptidoglycan, thereby additionally diminishing the antimicrobial effect of protamine [25,26,27]. On the P (125) DCPA disc, the concentration of calcium ions in the supernatant increased from about 1 μg·cm^−3^ to 1000 μg·cm^−3^. This was due to the dissolution of the DCPA powder (Figures S3 and S4). 

P (500) DCPA, which loaded the saturated level of protamine, released approximately 10% of the protamine from the disc into supernatants, which had a pH of around 6. A previous study demonstrated that protein adsorption to calcium phosphates, such as hydroxyapatite, employs multilayer adsorption. As a result of the increased distance as the number of layers increases, the electrostatic interaction between the protein molecules and the material surfaces became weaker [21]. Therefore, a level of protamine close to the saturated adsorption level may be easy to release. Taken together, the adsorbed protamine on P (500) DCPA released to the supernatant before bacterial growth and biofilm formation. Furthermore, the protamine remaining on the material surface also expressed antimicrobial activity. The antimicrobial activity of protamine adsorbed on DCPA powder could be sustained for long time.

## 5. Conclusions

In conclusion, this study demonstrated that protamine-adsorbed DCPA powders expressed antimicrobial activity against *S. mutans*. The mechanism of its antimicrobial activity followed two patterns: i) direct contact with bacteria and the material surfaces; and ii) releasing protamine to the surrounding environment. Additionally, the pH value affected the release of protamine and its antimicrobial activity. In particular, in conditions of low pH, more protamine was released from the materials at a higher rate. However, how protamine was released from materials in oral saliva, which contains high levels of ions and other proteins, is not clear. The mechanism of the adsorption and release of protamine therefore requires further investigation. Additionally, toxicity testing of protamine in oral tissue is also necessary.

Many kinds of dental materials, such as resin composites, contain calcium phosphates [28,29]. It is therefore expected that the application of protamine to various dental materials could prevent diseases caused by oral bacteria.

## Figures and Tables

**Table 1:** Characterization of protamine-loaded dicalcium phosphate anhydride (DCPA) powders.

Sample	Charged-Protamine	Loaded-Protamine	Zeta Potential	Median Size
	μg·cm^−3^	mg·m^−2^	mV	μm
P (0) DCPA	0	0	−22.34 ± 2.36	1.596 ± 0.166
P (125) DCPA	125	0.289 ± 0.022	2.95 ± 2.11	1.792 ± 0.157
P (500) DCPA	500	0.632 ± 0.003	19.02 ± 3.23	1.888 ± 0.271

## Supplementary Materials

The following are available online at https://www.mdpi.com/1996-1944/12/17/2816/s1, Figure S1: Adsorption isotherm of protamine, Figure S2: 3D architecture and cross section of biofilms on the disc, Figure S3: Calcium ion concentration of the supernatants after cultivation of *S. mutans* without sucrose, Figure S4: Calcium ion concentration of the supernatants after cultivation of *S. mutans* with 1% sucrose.
