# Supplementary material for: Antimicrobial Activity of Protamine-Loaded Calcium Phosphates against Oral Bacteria
Source: Materials (Basel). 2019 Sep 2;12(17):2816. doi: 10.3390/ma12172816 (PMC6747779; doi:10.3390/ma12172816)
Supplement: Supplementary file 1 [file materials-12-02816-s001.pdf]

materials

Antimicrobial Activity of Protamine-Loaded Calcium Phosphates against Oral Bacteria

Masashi Fujiki [1], Kodai Abe [1], Toru Hayakawa [2], TakatsuguYamamoto [3], Mana Torii [4], Keishi Iohara [5], Daisuke Koizumi [5], Rie Togawa [5], Mamoru Aizawa [1] and Michiyo Honda [1,*¶]

Supplementary Materials

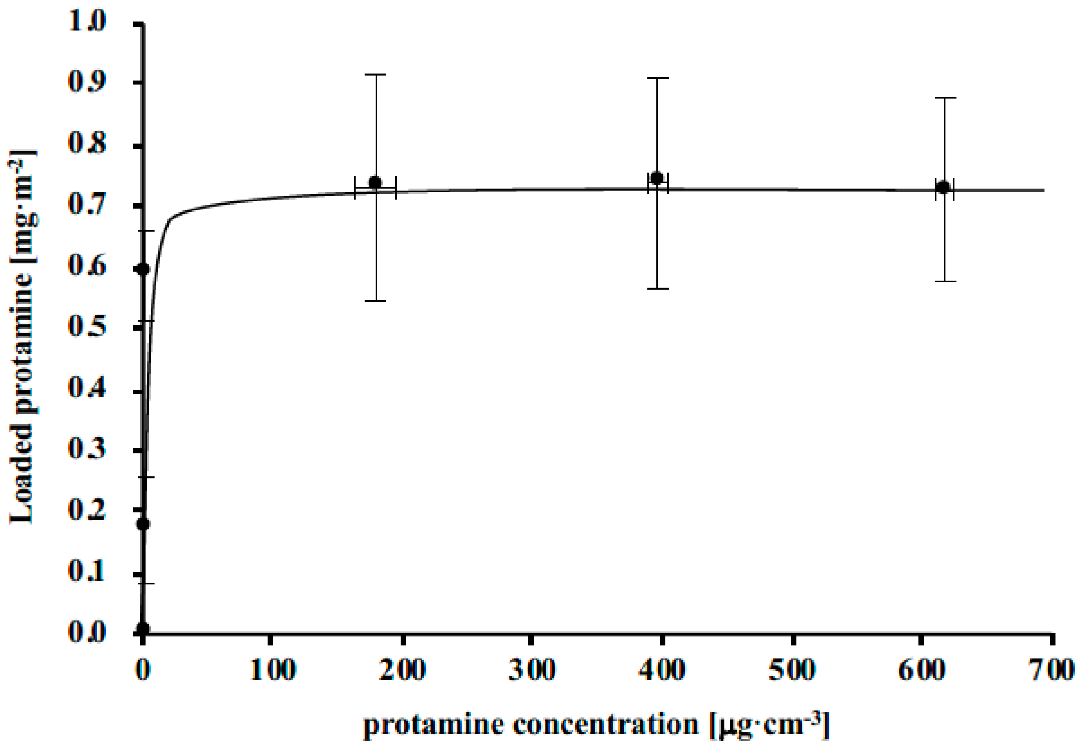

Figure S1. Adsorption isotherm of protamine. To determine the saturated level of adsorption of protamine, DCPA powders were immersed into various concentration (0, 100, 200, 400, 600, 800, 1000 μg·cm^{-3}) of protamine solution and rotated with a constant speed of 60 rpm for 48 h at room temperature. After adsorption of protamine, the samples were centrifuged for 15 min at 8000 rpm and the protamine concentration of the supernatants were measured by HPLC analysis. The powders were washed by ultrapure water 3 times and freeze-dried. The amount of loaded-protamine was measured by HPLC analysis.

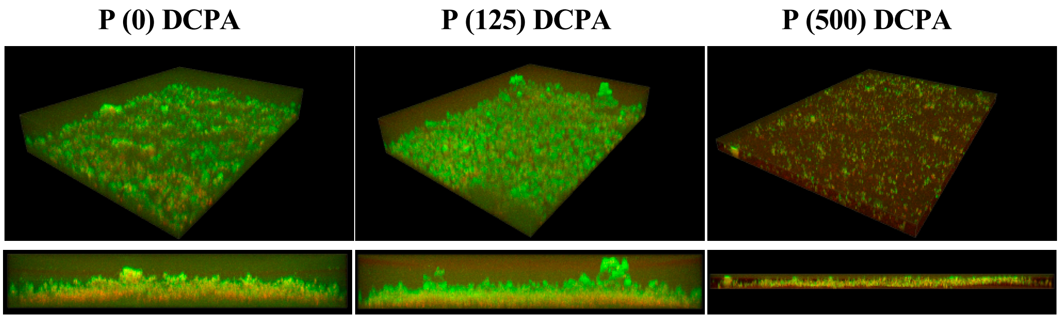

Figure S2. The 3D architecture and cross section of biofilms on the disc. After cultivation of *S. mutans* on the each disc with 1% sucrose, the biofilm were stained by SYTO9 and PI. Live bacteria appear green and dead bacteria appear red.

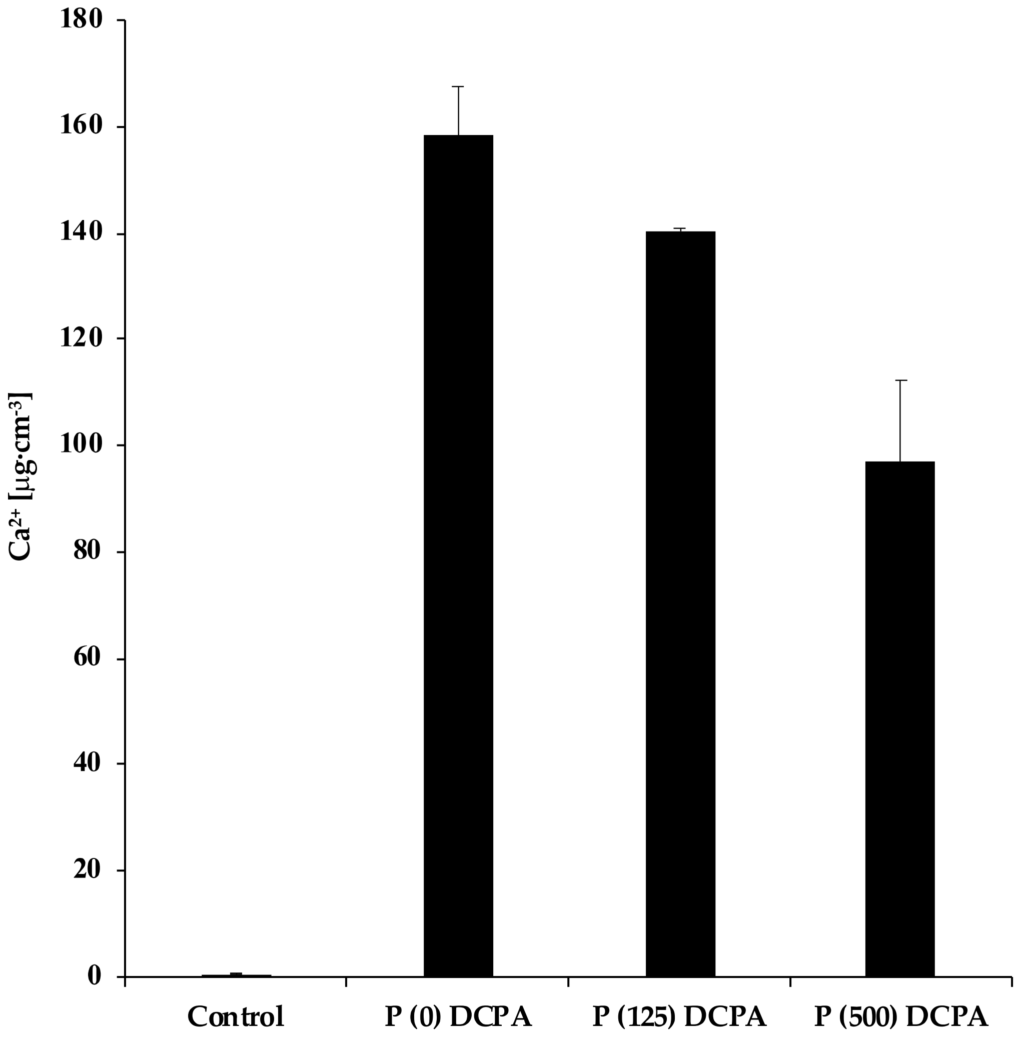

Figure S3. Calcium ion concentration of the supernatants after cultivation of *S. mutans* without sucrose. After cultivation of *S. mutans* on the discs of protamine-loaded DCPA under the cultivate condition without sucrose for 24 h, the calcium ion concentration of the supernatants was measured by ICP-AES.

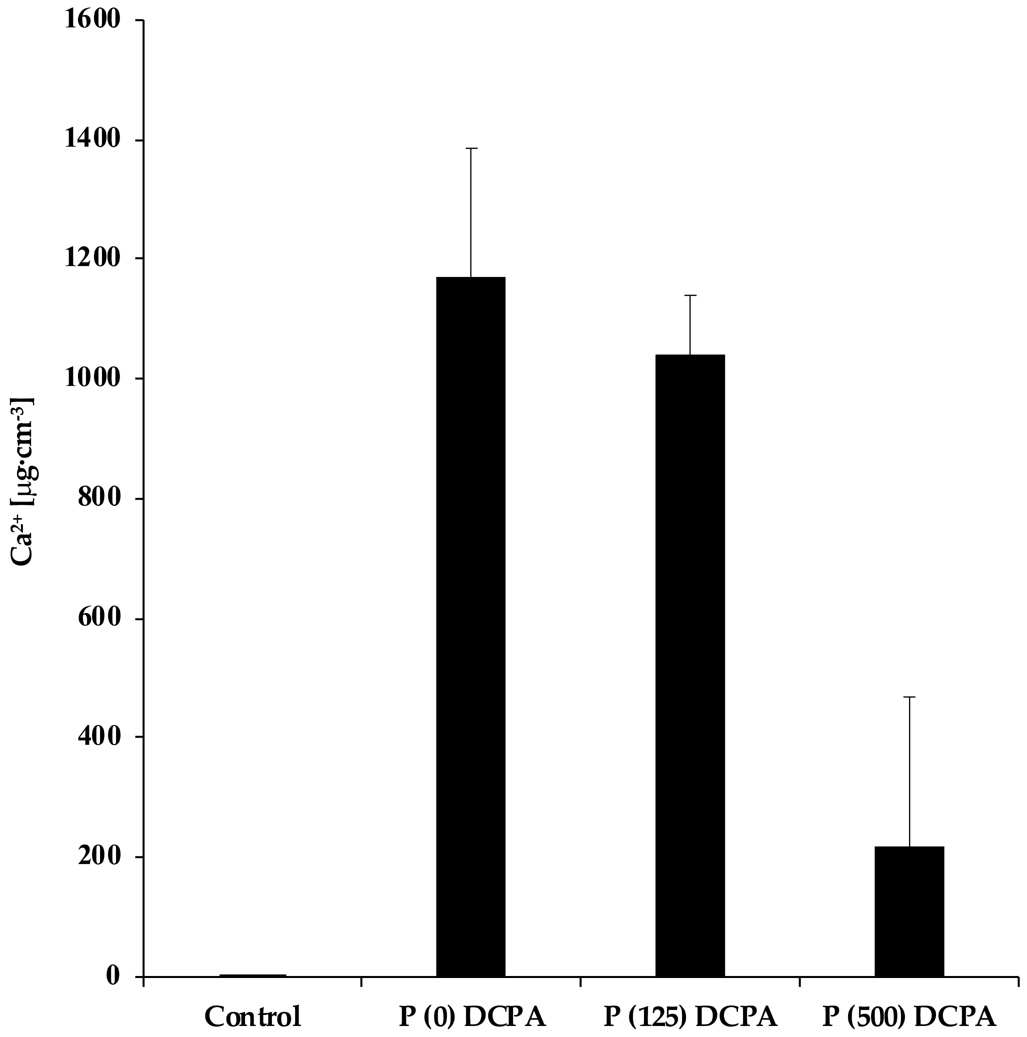

Figure S4. Calcium ion concentration of the supernatants after cultivation of *S. mutans* with 1% sucrose. After cultivation of *S. mutans* on the discs of protamine-loaded DCPA under the cultivate condition including 1% sucrose for 24 h, the calcium ion concentration of the supernatants was measured by ICP-AES.
